# Partially Reversible Bilateral Papilledema in a Patient Using Anastrozole: A Case Study

**DOI:** 10.7759/cureus.47925

**Published:** 2023-10-29

**Authors:** Joshua Wortsman, Ekaterina Proskuriakova, Barun Aryal, Sarah Khan, Danielle Sanchez, Kyle Anthony, Pam Khosla

**Affiliations:** 1 Internal Medicine, Mount Sinai Hospital, Chicago, USA; 2 Hematology and Oncology, Mount Sinai Hospital, Chicago, USA; 3 Internal Medicine, Ross University School of Medicine, Bridgetown, BRB

**Keywords:** bilateral papilledema, aromatase inhibitor, breast cancer, case report, anastrazole

## Abstract

Anastrozole is an endocrine-modifying agent used in the treatment of estrogen-sensitive breast cancer in the postmenopausal breast cancer population. Anastrozole is known for its side effect profile which includes an increased risk of osteoporosis. However, emerging evidence in the literature in the form of case studies demonstrates several potential ocular side effects due to the use of the medication. In our study, a 66-year-old female using anastrozole suffered severe bilateral papilledema that resolved after cessation of the medication. There is a growing body of evidence demonstrating the use of anastrozole and its impact on ocular health leading to deleterious side effects, such as papilledema.

## Introduction

Breast carcinoma is the most common form of malignancy affecting women in North America and approximately 25% of women with breast cancer eventually succumb to the disease [1]. About one-third of all breast cancers are estrogen-dependent and will respond to endocrine therapy by estrogen deprivation [1,2]. This response rate increases to 50% in postmenopausal women whose tumors are estrogen-positive [2]. The reduction of serum estrogen levels is an important tool for breast cancer treatment and has led to the development of multiple classes of drugs [1].

One such drug is anastrozole, a type II (non-steroidal) aromatase inhibitor (AI), and it is used for the targeted reduction of estrogen levels in postmenopausal women. Aromatase is the primary enzyme found in postmenopausal women responsible for the production of estrogen. This enzyme functions in aromatizing adrenal androgens such as androstenedione found in the peripheral tissues, subsequently converting them into estrogen; this process is stopped by an AI [2]. Evidence suggests anastrozole can inhibit aromatase activity by more than 96% and reduce plasma estrogen levels by greater than 78% following treatment [2]. Fundamentally, it has challenged tamoxifen as a possible first-line agent for postmenopausal patients with estrogen-sensitive breast cancer [1,2].

AIs like anastrozole, letrozole, and exemestane are widely utilized in postmenopausal women as adjuvant endocrine therapy for hormone-receptive breast cancer. While their adverse effects are well-documented, ocular complications, particularly those related to anastrozole use, remain relatively scarce in the literature.

In this case report, we aim to explore the limited existing data on ocular issues associated with AIs and, in particular, the rarely reported complication of papilledema, with the goal of increasing awareness regarding the ocular adverse effects of these drugs. Unlike tamoxifen, which has well-established ocular side effects, AIs pose a unique challenge [3]. Studies have identified retinopathy issues such as crystalline retinopathy, hemicentral retinal artery occlusion, and retinal hemorrhages associated with these third-generation AIs. Furthermore, ocular surface conditions, including corneal epithelial changes, blepharitis, and keratitis, have been observed in patients taking AIs [4]. Although these ocular side effects are likely infrequent, we emphasize the importance of maintaining a high level of clinical suspicion when evaluating patients who experience visual symptoms while on AIs. It is our contention that larger prospective studies are essential to gain a more comprehensive understanding of these ocular complications and their management. 

## Case presentation

**Figure 1:**
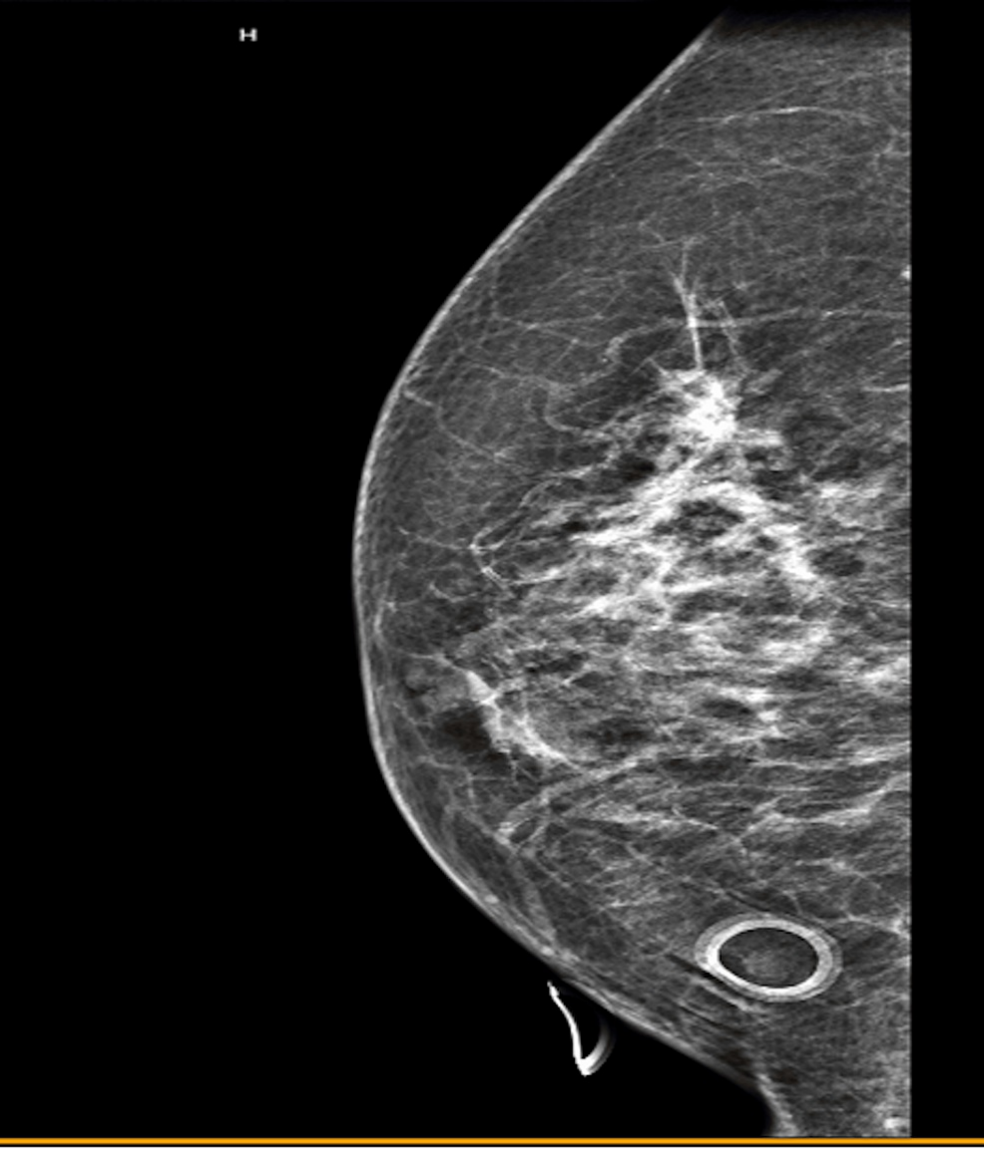
Diagnostic mammogram revealed irregularities on the right breast.

A 66-year-old female presented to our clinic after a positive routine mammogram (Figure 1). On biopsy, she was found to have ductal carcinoma in situ with both cribriform and solid type in her right breast, which was estrogen receptor (ER) 98% positive, (progesterone receptor) PR 2% positive, and human epidermal growth factor receptor 2 (HER2) negative. 

**Figure 2:**
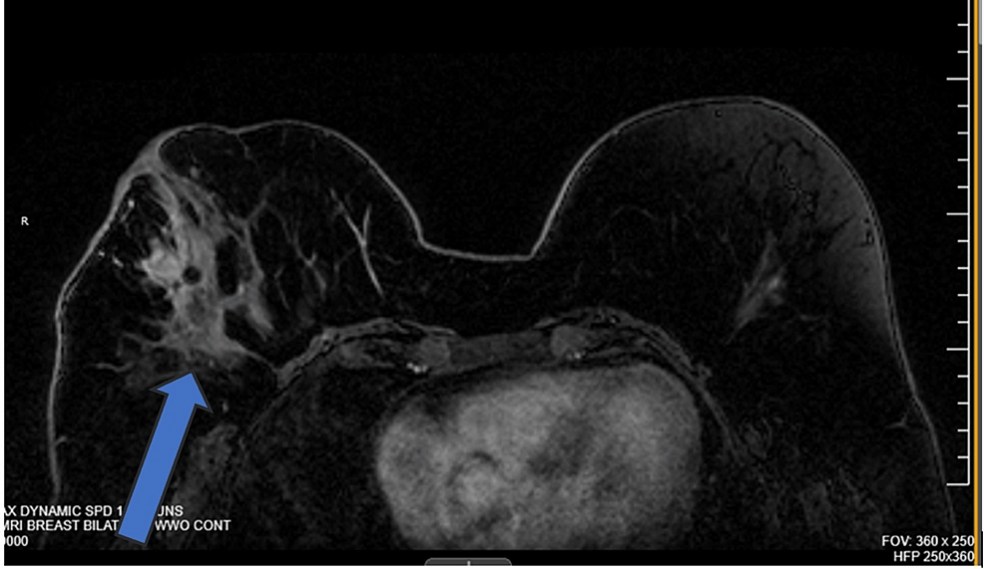
MRI of the bilateral breast with and without contrast showing an irregular mass on the right breast.

Magnetic resonance imaging (MRI) confirmed a mass located at the outer quadrant of the right breast with irregular margins and shapes correlating to the area of the biopsy (Figure 2). The left breast was biopsied for a retro-areolar 1 cm mass founded on imaging to be benign ductal hyperplasia and microcalcifications.

The patient elected for a right breast mastectomy yielding multifocal invasive ductal carcinoma grade 2, measuring 1 cm, and multifocal invasive lobular carcinoma stage 1 with pathology staging of pT2, pN0. All the margins were negative for infiltrating lobular carcinoma, intraductal carcinoma (IDC), and ductal carcinoma in situ (DCIS), and all four lymph nodes were negative. The surgical report indicated that the patient had an Oncotype Dx recurrence score of 0 with a 3% distant recurrence at nine years and a 1% added benefit from additional hormonal therapy. During this time, the patient elected hormonal treatment, which consisted of anastrozole and cholecalciferol.

One month after starting the anastrozole at a daily dose of 1 mg, the patient presented to the emergency department, endorsing right eye vision loss for 10 days. The vision loss started as dots and gradually worsened, resulting in the loss of the bottom half of vision in the right eye. She first visited an optometrist who diagnosed her with severe papilledema and referred her to the emergency department. On the physical examination, her pupils were equally round and reactive to light, the right eye had significant vision loss, extraocular movements were intact, and there was clear discharge from her right eye. There were no focal neurological deficits on examination. Visual acuity (VA) was 20/100 20/25 OD/OS. Fundus examination revealed 1+ optic disc edema OD/OS without cotton wools. Otherwise, the patient's physical exam was within normal limits, and she denied experiencing head or neck pain, nausea, vomiting, pain in her eyes, or palpitations. Imaging modalities such as computed tomography (CT) and MRI of the brain revealed no acute findings. A lumbar puncture was performed, revealing a normal opening pressure and no significant findings in cerebrospinal fluid. The laboratory results for erythrocyte sedimentation rate (ESR) and C-reactive protein (CRP) were within normal ranges. During her hospital course, the hormonal therapy was discontinued, and her vision gradually began to improve, seeing gray rather than black. In the outpatient setting, a magnetic resonance (MR) venogram was ordered, and it was negative for any acute findings. An MR angiography and MRI of her orbits were performed, both of which were normal.

After discontinuing anastrozole for approximately two months, the patient's vision resolved to some extent. While in the hospital, she had limited ability to see from her right eye; two months after cessation of anastrozole, the patient's vision began to improve, allowing her to see outlines without greater detail.

## Discussion

In our case study, we describe a patient who developed reversible papilledema with partial recovery as a consequence of using anastrozole. Our patient presented to the emergency department with severe papilledema, and after cessation of the usage of anastrozole, her visual symptoms improved over two months in the outpatient setting. The incidence of ocular side effects from the use of anastrozole or other AIs is rare but documented within the literature, describing various ocular pathologies and symptoms. The literature further displays that ocular complications linked to the use of anastrozole are slightly more prevalent compared to other AIs [4].

Papilledema is the swelling of the optic disc due to increased intracranial pressure but may not correlate directly to the level of intracranial pressure (ICP). Visual loss from papilledema may occur even in cases of mild elevation of ICP through a variety of pathophysiological mechanisms but primarily from direct compression of the visual pathway [5]. Coppes et al. published a case of papilledema and macular edema secondary to the use of anastrozole [6]. They proposed that changes in estrogen levels may have led to vascular permeability and optic nerve swelling. In their case, the patient experienced near-complete resolution of swelling and vision loss in the affected eye with a course of prednisolone and by stopping the usage of anastrozole [6]. It should be noted that letrozole, another commonly used type II AI, has also been reported to cause macular edema [4].

Estrogen receptors are found widely in the body, including on vascular smooth muscle, endothelial cells, and the retina. It has been shown that peak systolic and diastolic velocities of the central retinal artery increase with the intranasal administration of 17 beta-estradiol [7]. Therefore, estrogen depletion might affect appropriate retinal perfusions and vascular permeability, leading to maculopapular edema. Additionally, in a case report by Karagoz et al., it was suggested that estrogen depletion from anastrozole may affect the retinal vascular system, increasing the risk of thromboembolism in the ocular system leading to retinal hemorrhage, edema, and central retinal artery occlusion [7].

In addition to possible vascular permeability caused by estrogen deprivation, it is also known that both alpha and beta estrogen receptors are expressed and functionally active in the human retinal pigment epithelium, the neurosensory retina, and the choroid providing neuroprotection to the retinal cells [8]. The use of AIs such as anastrozole deprives these receptors of estrogen and diminishes their protective effects. The downstream effects of estrogen deprivation may contribute to vascular changes leading to macular edema and retinal pathology in patients using AIs. 

Several case reports found in the literature have discussed the potential ocular effects of AIs and the management of these side effects. For instance, Coppes et al. detailed a single case involving anastrozole usage, which led to severe bilateral optic disc swelling and impaired visual acuity; notably, these symptoms almost completely resolved after discontinuing anastrozole [6]. Sathiamoorthi et al. described a case related to anastrozole, where a patient developed bilateral cystoid macular edema and uveitis [9]. This condition was successfully managed using difluprednate ophthalmic emulsion drops, and the patient continued taking anastrozole [9]. Moschos et al. reported a single instance in which letrozole use resulted in unilateral macular edema, and this issue improved following intravitreal injection of ranibizumab [8]. Turaka et al. found that 41 women taking AIs exhibited various ocular issues, with 73% experiencing blepharitis, 29% having poor tear function, 22% showing conjunctival injection, and 29% presenting with superficial keratitis [10].

It's worth noting that there are currently no established guidelines for the screening and management of these adverse effects. As a result, oncologists should remain vigilant, being attentive to potential ocular side effects associated with the medication, recognizing early warning signs, and guiding patients toward the appropriate diagnostic and treatment procedures.

## Conclusions

Visual disturbances associated with the use of aromatase inhibitors (AIs), specifically anastrozole, are variable and can present in various ways. Our case study is, to our knowledge, the second to demonstrate reversible papilledema with vision loss in the context of using anastrozole. Given that AIs are considered the standard of care of adjuvant endocrine therapy in postmenopausal women, it is essential to further understand their potential side effect profile. When treating patients using AIs and anastrozole, a high index of suspicion should be kept in mind when addressing new ocular symptomatology.

## References

[1] Buzdar AU, Robertson JF, Eiermann W, Nabholtz JM (2002). An overview of the pharmacology and pharmacokinetics of the newer generation aromatase inhibitors anastrozole, letrozole, and exemestane. Cancer.

[2] Wiseman LR, Adkins JC (1998). Anastrozole: a review of its use in the management of postmenopausal women with advanced breast cancer. Drugs Aging.

[3] Yoon CI, Lee HS, Jeon S, Kim D, Park WC (2023). Relationship between tamoxifen and cataracts: a nationwide cohort study of women in South Korea. Breast Cancer Res Treat.

[4] Almafreji I, Smith C, Peck F (2021). Review of the literature on ocular complications associated with aromatase inhibitor use. Cureus.

[5] Schirmer CM, Hedges TR 3rd (2007). Mechanisms of visual loss in papilledema. Neurosurg Focus.

[6] Coppes OJ, Lukas RV, Fleming GF, Nichols J, Tenney M, Bernard J (2014). Bilateral optic disc swelling following anastrozole therapy. Neuroophthalmology.

[7] Karagöz B, Ayata A, Bilgi O (2009). Hemicentral retinal artery occlusion in a breast cancer patient using anastrozole. Onkologie.

[8] Moschos MM, Chatziralli IP, Sergentanis T, Zagouri F, Chrysikos D, Ladas I, Zografos G (2016). Electroretinographic and optical coherence tomography findings in breast cancer patients using aromatase inhibitors. Cutan Ocul Toxicol.

[9] Sathiamoorthi S, Ruddy KJ, Bakri SJ (2018). Association of uveitis and macular edema with anastrozole therapy. JAMA Ophthalmol.

[10] Turaka K, Nottage JM, Hammersmith KM, Nagra PK, Rapuano CJ (2013). Dry eye syndrome in aromatase inhibitor users. Clin Exp Ophthalmol.

